# Deep Ensembles Are Robust to Occasional Catastrophic Failures of Individual DNNs for Organs Segmentations in CT Images

**DOI:** 10.1007/s10278-023-00857-2

**Published:** 2023-06-08

**Authors:** Yury Petrov, Bilal Malik, Jill Fredrickson, Skander Jemaa, Richard A. D. Carano

**Affiliations:** Genentech, Inc., 1 DNA Way, South San Francisco, CA 94080 USA

**Keywords:** Automated organ segmentation, Computed tomography, Deep neural networks, Deep ensembles

## Abstract

Deep neural networks (DNNs) have recently showed remarkable performance in various computer vision tasks, including classification and segmentation of medical images. Deep ensembles (an aggregated prediction of multiple DNNs) were shown to improve a DNN’s performance in various classification tasks. Here we explore how deep ensembles perform in the image segmentation task, in particular, organ segmentations in CT (Computed Tomography) images. Ensembles of V-Nets were trained to segment multiple organs using several in-house and publicly available clinical studies. The ensembles segmentations were tested on images from a different set of studies, and the effects of ensemble size as well as other ensemble parameters were explored for various organs. Compared to single models, Deep Ensembles significantly improved the average segmentation accuracy, especially for those organs where the accuracy was lower. More importantly, Deep Ensembles strongly reduced occasional “catastrophic” segmentation failures characteristic of single models and variability of the segmentation accuracy from image to image. To quantify this we defined the “high risk images”: images for which at least one model produced an outlier metric (performed in the lower 5% percentile). These images comprised about 12% of the test images across all organs. Ensembles performed without outliers for 68%–100% of the “high risk images” depending on the performance metric used.

## Introduction

Deep ensembles [1], inspired by the bootstrap (bagging) [2], boosting [3], and random forest [4] approaches, aim at combining the statistical power of diverse solutions explaining the same data. It has been empirically observed that training the same network multiple times, each time initializing it with random weights, and then averaging the solutions, consistently bests a single model’s performance, on average. Aside from the simple averaging of the ensemble’s solutions, followed by a thresholding operation (label-voting scheme [5]), more sophisticated combinations of the solutions are possible. For example, in the case of image segmentation, a particular type of the label-voting scheme was proposed, where individual ensemble segmentations as well as averaged segmentations at different voting thresholds are compared with the help of a linear regression model trained on their pairwise Dice scores and individual Dice scores with the ground truth segmentations [6]. This approach showed some performance improvement when compared to single-model performances. The STAPLE algorithm [7] is an example of weighted averaging of individual solutions that is particularly popular in the field of medical imaging, where several “ground truth” segmentations from different readers need to be combined into one.

While the Deep Ensembles approach was originally proposed and tested for classification tasks, it has since been applied for image segmentation, e.g., optic cup and disk [8], white matter lesions [9], cervical cancer lesions [10], brain tumors [11], infant brains [12] and livers [13]. Within the image segmentation context, Deep Ensembles were also used to train on small datasets [14], automatically ensemble the two highest-performing models [15], estimate the uncertainty of medical image segmentations [12, 16, 17] as well as to address the issue of disagreement in ground truth segmentations from multiple readers [18].

These studies showed that ensembles consistently improved segmentation accuracy over single models, where the accuracy metric was averaged over multiple images in test datasets. Yet, in the context of safety-critical applications, medical applications in particular, the *robustness*, i.e., the reliability of an algorithm when applied to each individual case also plays a very important role. One can even argue that some guarantee that an algorithm never fails catastrophically can be traded for a slight decrease in its average performance. Deep Ensembles promise a viable solution to the robustness requirement, because they implement an approach, where different versions of the solution potentially have independent failure points. Hence, combining these by means of the majority vote or a similar scheme should result in a more reliable solution [19]. [11] argued along the same lines and demonstrated that ensembles of various types of neural networks were characterized by better performance metrics than a number of individual models in the brain tumor segmentation task.

While many studies have shown that ensembles provided superior *averaged* performance metrics, to the best of our knowledge no studies explicitly demonstrated their superior robustness. Robustness is particularly valuable in medicine, where bad errors can be very costly, and given that DNNs generally score poorly on robustness, Deep Ensembles can play a more important role here than a mere improvement of average performance.

The purpose of this study was to explore the Deep Ensembles approach for medical image segmentation and their effect on the segmentation robustness. We chose organ segmentation in CT images as the testing ground, but it is likely that our findings apply to other types of segmentation tasks in medical images and to image segmentation in general. Segmentation of internal organs in medical images is a common task required for identifying organs affected by a disease, computer-assisted surgery, radiotherapy, volumetric measurements for group and population statistics, etc. In particular, The Response Evaluation Criteria in Solid Tumors (RECIST) standard of cancer burden [20] stipulates at most 2 “target” lesions per organ, which necessitates assigning each of the target lesions to an organ. Therefore, automating the RECIST metric requires segmentation of all the organs where such lesions may be found. Occasionally, this is a challenging task even for a human reader because nearby tissues form no clear boundaries for many organs, especially, if no contrast agent was used. Poor image resolution, acquisition artifacts, pathological tissue appearance, and varying imaging protocols pose additional problems. While for some organs (e.g., liver, lungs, kidneys) the segmentation accuracy (the Sorensen-Dice coefficient or, simply, the Dice score, *DSC*, in the rest of the paper) reaches over 95% on average, for other organs (e.g., pancreas, adrenal glands, intestines, uterus, prostate) the metric is typically much lower.

## Material and Methods

We used a large dataset of labeled organ segmentations in contrast-enhanced CT images to investigate the use of Deep Ensembles for organ segmentation. The V-Net architecture [21] was chosen for all ensemble models, while the number of models in an ensemble, models hyperparameters, and the different ways to combine individual solutions within an ensemble were varied systematically to study their effect on the accuracy and the robustness of organ segmentations.

### Data and Preprocessing

Predominantly contrast-enhanced CT images were used in the present study. While most images were in the portal venous phase with some normal variation, some of the images came from publicly available CT databases, where a mixture of contrast phases was present. Also, a small proportion of the used in-house images were either in the Arterial contrast phase or not contrast-enhanced (see Table 1 for details). Hence, all contrast uptake phases were represented in the dataset and were not discriminated against during training or testing. The dataset comprised in-house images from several phase III clinical trials as well as several publicly available datasets (TCIA [22], BTCV [23], CT-ORG [24], Medical Decathlon [25]). The in-house (anonymized) dataset used in this study was a small subset of CT images from five global, multicenter, open-label phase 3 clinical trials in metastatic nonsquamous non-small-cell lung cancer (NSCLC), follicular lymphoma, and HER2-positive advanced breast cancer from patients who had not previously received chemotherapy. The trials received institutional review board (IRB) approval, including for secondary data usage of anonymized data.

The training dataset comprised all relevant images from the publicly available datasets (TCIA, BTCV, CT-ORG, Medical Decathlon) and a subset of images from several large in-house clinical trials called IMpower150 (ClinicalTrials.gov number NCT02366143 [26]), GOYA (ClinicalTrials.gov number NCT01287741 [27]) and MARIANNE (ClinicalTrials.gov number NCT01120184 [28]). The test dataset was a subset of images from a separate in-house clinical trial called IMpower131 (NCT02367794 [29]). This dataset had been allocated for testing before any training was done and was never used for training, validation, or hyperparameter tuning to avoid any “peeking” artifacts [30]. The ground truth segmentations were done by a team of trained radiologists, each image has been segmented by one radiologist only, and the results were quality checked and adjudicated if necessary.

The anatomical coverage and image resolution varied. Only full-torso images with better than 2 mm in-plane and 5 mm (axial) slice thickness and no acquisition artifacts were chosen for the in-house dataset. Otherwise, the images were chosen at random. The following 21 organs were segmented for these images: right lung, left lung, mediastinum, liver, right adrenal gland, left adrenal gland, stomach, spleen, gallbladder, right kidney, left kidney, pancreas, aorta (abdominal section), small intestine, large intestine, bladder, right breast (fibroglandular tissue and fat tissue), left breast, uterus, prostate, and bones (not discriminated into individual bones).

**Table 1:** Distribution of all images in the in-house database over the gender of the participants and the contrast phase of the CT acquisition. Gender data were missing for some of the images. More details on the participants of the in-house studies can be found in the respective references

**Organ**	Study	Total	Females	Males	Portal venous	Arterial	No contrast
Uterus & Breasts	MARIANNE IMPower150 IMPower131	267	267	0	208	24	35
Prostate	IMPower150 IMPower131	133	0	133	116	15	2
Aorta & Intestines	GOYA IMPower150 IMPower131	227	110	64	180	18	29
The rest of the organs	IMPower150 IMPower131	200	67	133	181	17	2

There were four test datasets used: one for the male-only organs (prostate, 37 images), one for the female-only organs (breasts and uterus, 37 images), one for the pancreas (78 images) and one for all other organs (38 images). Pancreas evaluation received more test images because it was one of the hardest organs to segment. The training datasets were comprised of the remaining in-house images combined with additional images from the publicly available datasets (TCIA, BTCV, CT-ORG, Medical Decathlon). Since all organs used in our study were not present in all publicly available datasets, the number of training CT images varied from 162 to 900 depending on the organ. 16% of the training images were allocated for validation and images in this subset could vary from model to model. We tested whether using different validation subsets across the Deep Ensemble models increased the ensemble’s performance, but did not find any clear benefit compared to using the same validation dataset for all models, hence the same validation subset was used for all models within a given organ’s ensemble.

Image preprocessing consisted of the following steps:

- resizing original CT images to 1 $mm^3$ resolution
- cropping to a 192x192x192 fragment comprising the entire organ with respect to its middle point along each dimension
- de-noising the resulting image
- rescaling the image intensities: first clamping to [**-**1000 1500] Hounsfield units (HU) and then linearly mapping to the [0 1] interval.
- augmenting images by random affine transformations ($\le 5^\circ$ 3D rotation, $\le 5$% scaling, $\le 5$% shear, $\le 10$ voxels translation)

In the resizing step, cubic spline interpolation was used for CT images and nearest-neighbor interpolation for the binary segmentation masks. In the cropping step, the cubic spline down sampling was occasionally used for larger organs in order to fit the whole organ into the crop box. We tried two variants of the de-noising step: a median filter with 3x3x3 window and a nonlinear mean filter [31] implemented by the Python’s skimage package denoise_nl_means function (patch size and distance set to 3, sigma set to 0.005). The latter filter achieves noise removal on par with the simple median filter while largely preserving the sharpness of edges in the images. For all organs, where both de-noising filters were tried, the segmentation performance was the same or better for the nonlinear mean filter, and, thus, input images were de-noised in this way for the rest of the study. The augmentation step was done prior to training to minimize the training time. The original image and 1–7 of its augmented copies (depending on the number of the training images for a given organ) were used for training and validation bringing the total number of training images to 1500–2000 per organ. Original images without any augmentations were used for testing.

### Neural Network and Training

The V-Net network architecture was chosen for this study. The V-Net was first introduced for 3D medical image segmentation and showed a superior performance in this task [21]. We also tested the U-Net [32] and the Dense V-Net [33] architectures, but their performances were found to be inferior to the V-Net, at least in the range of hyperparameters that we explored for this project. Since the research focus of our study was on the use of Deep Ensembles for segmentation, the precise network architecture probably had little effect on the main findings.

The V-Net architecture was identical to that in the original paper, including the number of stages (5) and the number of channels at each stage (16, 32, 64, 128, 256), but we found that 3x3x3 kernels performed on par with 5x5x5 kernels used in the original model, so we used the smaller kernels to ease memory requirements. Other hyperparameters, including the dropout rate, 0.25, the Adam optimizer learning rate, 0.0002, and beta value, 0.5, were set based on a relatively exhaustive hyperparameter space exploration performed for the pancreas segmentation task. ReLU activation functions were used throughout the network, except for the final sigmoid transformation of the single channel in the output layer.

A separate ensemble of V-Nets was trained for each organ. We tried using different activation functions and dropout rates across models within each ensemble to instigate more diverse solutions, but these ensembles performed slightly poorer than ensembles composed of models with the same (optimal) hyperparameters and activations. Hence, the same exact V-Nets were used for all models, each network trained from a randomly initialized state using the He normal initialization [34]. Given the large image size, each model was trained in batches of 8 images using 8-GPU node instances, i.e., one image per GPU. Although the Tensorflow’s SyncBatchNormalization was used to synchronize batch normalization among multiple GPUs, one might expect that batch normalization was still noisy due to the small batch size. The loss function was defined via the Dice score as $1 - DSC$, i.e., *DSC* was maximized during training. For every 10 epochs, the Dice score was calculated for the validation dataset, and the training continued until the slope of the line fit for the last seven Dice scores fell below 0.001 indicating saturation of the validation score. Typically, 120–160 epochs were required for the saturation to occur.

### Stochastic Weights Averaging (SWA)

Another ensembling approach termed Stochastic Weight Averaging [35] argues that once training of a model more or less converged it is beneficial to sample the space around the solution using Stochastic Gradient Descent (SGD), with a relatively large learning rate, all the while averaging the resulting samples in the weight space. The authors hypothesize that this procedure samples from a hyper-sphere surrounding the “true” optimum and hence the samples’ average brings the solution closer to the optimum. Since weight averaging only makes sense while averaging solutions belonging to the same local minimum of the loss function, the SWA ensemble effectively exploits only one local minimum. In contrast, Deep Ensembles, where models predictions are averaged instead of their weights, are capable of exploiting multiple local minima hence potentially capturing a richer palette of relevant features. We tested the SWA ensembling for pancreas segmentation. For each of the 16 pancreas models previously trained, we continued training with the following SWA procedure: Starting from the trained weights, the SGD was applied for 30 epochs. Following each epoch, the model weights were averaged over all the epochs finished so far. We observed that the corresponding validation scores typically converged after 15–20 epochs. The epoch with the highest validation score indicated the optimal number of SWA samples, over which the weights of the final model were averaged. Several values of the SGD learning rate, *lr*, within the [0.0001, 0.05] interval were tested for one of the models; the best performance was achieved for $lr = 0.001$. This value was then used for SWA training of all the models.

### Distillation of Deep Ensembles

Performance of Deep Ensembles can be matched by single models after training on the averaged outputs of the ensembles instead of the actual ground truth labels, the so-called distillation technique [36]. Using a single-distilled model instead of an ensemble would save an order of magnitude in memory and computational resources (or execution time). The distillation technique has been shown to be successful in classification tasks, and we wanted to test it for organ segmentations. To this end, synthetic ground truth segmentations for the training dataset images were created by running 10 ensemble models on the images and averaging over their predictions along with the actual ground truth segmentation, all with equal weights. For the pancreas, we continued the distillation process from single distillation to double distillation by training a set of models on averaged segmentations of the distilled models. The same V-Net architecture was used for distilled models as was used for regular models. Multiple models were trained starting from random initialization parameters to form Deep Distilled Ensembles because we wanted to see if such ensembles showed improvements over a single-distilled model.

### Evaluation Metrics

Because the Dice score was maximized during training it was also used as the principal evaluation metric for organ segmentation accuracy. In some cases we additionally calculated and plotted three other popular metrics [37, 38]: the Relative Volume Difference, *RVD*, the Average Symmetric Surface Distance, *ASSD*, and the Maximum Symmetric Surface Distance, *MSSD*. While the Dice score gives relative overlap between the ground truth and model segmentation volumes, the *RVD* provides just the relative agreement between the volumes ignoring their overlap. The *ASSD* averages all the nearest distances from points on the boundary of the model segmentation to the boundary of the ground truth, and vice versa, while *MSSD* gives the greatest of these distances (the Hausdorff distance between the two boundaries).

## Results

### Probability Threshold for Ensemble Averaging

**Fig. 1:**
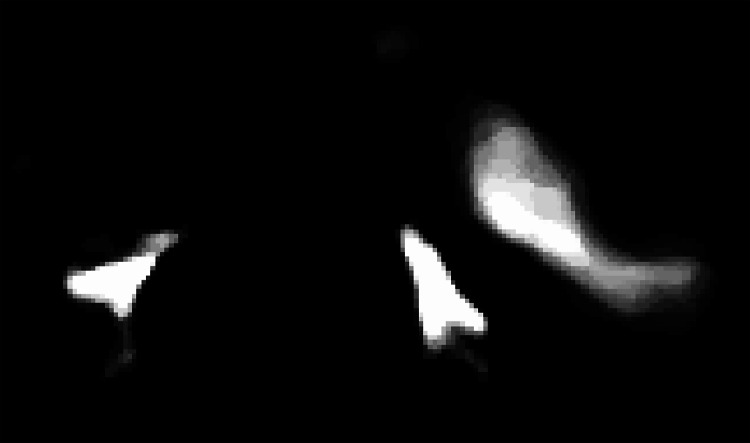
Averaged segmentations of pancreas and adrenal glands produced by multiple models (a representative axial slice is shown). The resulting distributions can be interpreted as probabilities of a voxel belonging to a given organ

Most commonly, the Deep Ensemble solution $S_{ens}$ is formed by averaging solutions $S_m$ of all individual ensemble models *m*: $S_{ens} = \frac{1}{m}\sum _m S_m$. This is illustrated in Fig. 1, where segmentations of the pancreas and two adrenal glands, averaged over multiple models, are shown for each of the organs. While for classification tasks this involves no additional parameters (one just picks the category with the highest resulting probability), segmentation typically requires a binary mask as its output, and the averaged output masks of ensemble models need to be thresholded at some probability value $p_{thr}$. The choice of the probability threshold determines how conservative the ensemble’s predictions are: probability 1 corresponds to all of the ensemble masks agreeing for a given voxel, while lower thresholds allow for progressively stronger disagreements.

**Fig. 2:**
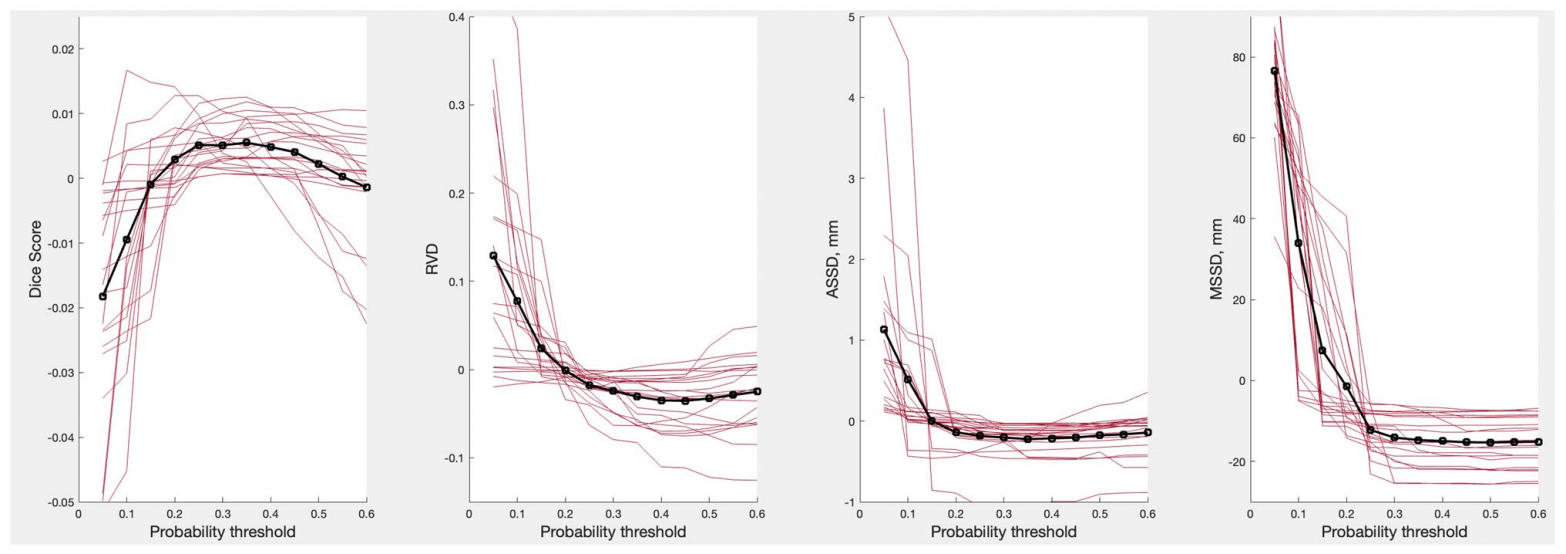
Dice score and *RVD*, *ASSD*, *MSSD* metrics for all 21 organs vs. $p_{thr}$. Note that the mean for each curve was set to 0 for illustrative purposes, hence the y-axes display only relative numbers. The metrics for individual organs are shown by red curves, the metrics averaged over all the organs are shown by black curves

We treated the probability threshold as a hyperparameter and plotted the resulting Dice scores and *RVD*, *ASSD*, *MSSD* metrics for the test datasets as a function of $p_{thr}$ in Fig. 2. Thresholds maximizing the ensembles’ Dice score were between 0.2 and 0.4 for most organs and 0.35 when averaged over all organs. Thresholds maximizing *RVD*, *ASSD*, *MSSD* metrics averaged over all the organs were 0.45, 0.35 and 0.5 respectively. Note that the averaged metrics changed only slightly for $p_{thr} > 0.25$, hence the exact choice of the $p_{thr}$ value was of little importance as long as it was above 0.25. Since the Dice score was used for model training we set $p_{thr}$ to 0.35 for the rest of the study. Interestingly, repeating the same for the validation dataset shifted the optimal value to 0.4, while for the training dataset the value was 0.5. This apparent increase of the optimal threshold along with the increase of the amount of information about images available during training might be lacking statistical significance and was left for a future investigation. For the present purposes, we note that the effect of varying $p_{thr}$ above 0.25 was negligible and did not change the results of this study.

### Ensemble Size

**Fig. 3:**
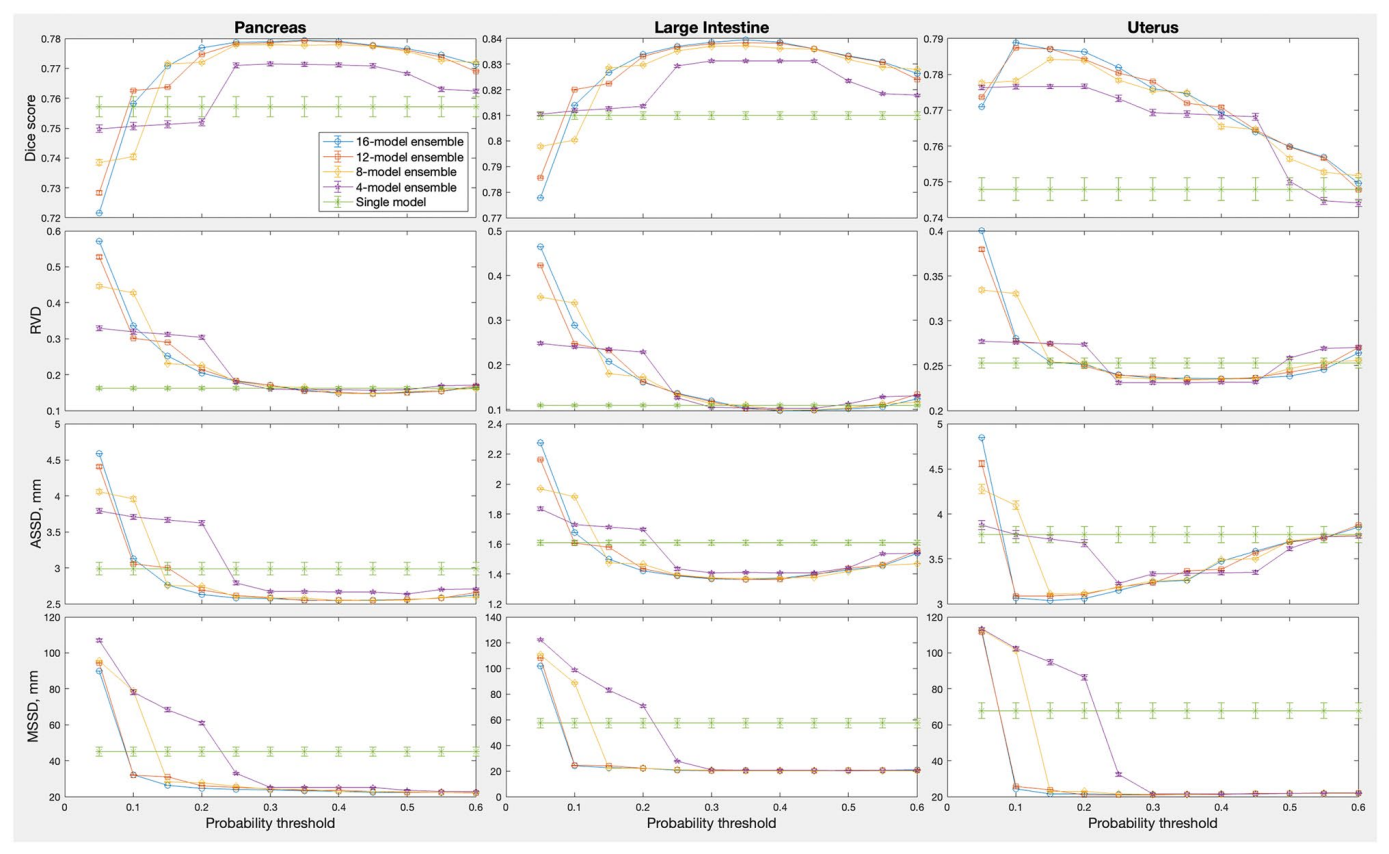
Effect of ensemble size and probability threshold for the pancreas, large intestine and uterus ensembles. Here, the ensemble size was 16; results for the smaller ensemble sizes were averaged over 10 random samples (without repetition) from the full ensemble. Error bars indicate one standard deviation of the mean over the random subsamples

The ensemble sizes in Fig. 2 varied from 8 to 16 models, depending on the organ. We also varied the number of models for the same organ to see if there was any effect on the optimal $p_{thr}$. Results for the three organs, large intestine, uterus and pancreas, for which 16 models were trained for each ensemble, are shown in Fig. 3. The ensemble size had no consistent effect on the optimal $p_{thr}$: the Kruskal-Wallis test for the optimal threshold value vs. ensemble size gave probability of the null effect at 0.270, 0.864 and 0.002 for pancreas, large intestine and uterus respectively when using the *DSC* metric, and similarly inconsistent probabilities for the other three metrics. The best performance metrics, on the other hand, improved monotonically with the ensemble size ($p_{null} =$ 0.006, 0.0002, 1e-5 respectively for the three organs using the Kruskal-Wallis test on the *DSC* metric), quickly saturating near 8–10 models per ensemble.

### Combining Ensemble Segmentations

One might wonder if simple averaging of ensemble’s segmentations is the best solution. For example, [6] suggested calculating Dice scores of a given model’s segmentation *S* with all other models segmentations (excluding itself), $DSC( S^m, S^n)$, where *m* and *n* enumerate ensemble models, as well as with the ground truth, $DSC( S^m, S^{GT})$, using all the training data, and then fitting a linear regression, $DSC( S^m, S^{GT}) = \alpha _m + \sum _n \beta _{mn} DSC( S^m, S^n)$ for each model *m*. The fit is then used for a new image to estimate the Dice score of each model’s segmentation with the unknown ground truth, $DSC(S_{new}^m, S_{new}^{GT})$, the segmentation *m* with the highest score to be used as the best segmentation.

**Fig. 4:**
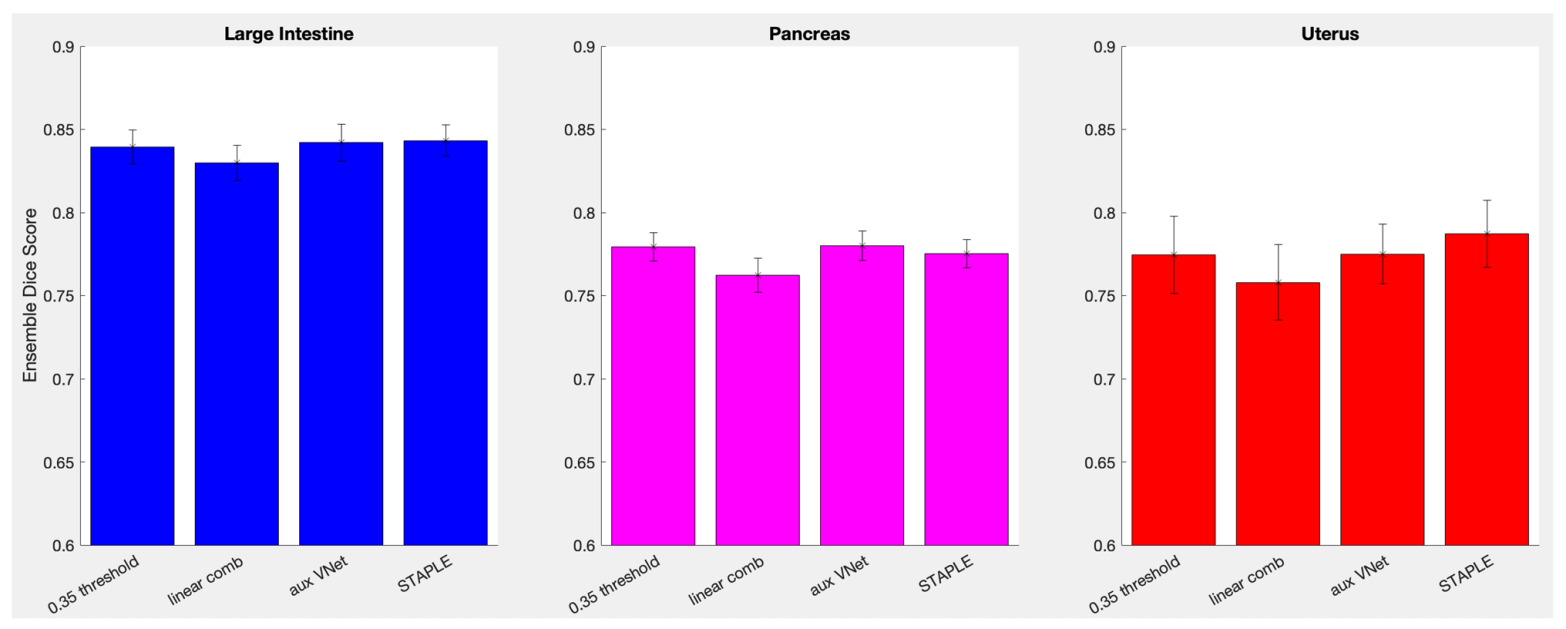
A comparison of different modes of combining ensemble segmentations for large intestine, pancreas and uterus

Instead of averaging all segmentations with equal weights or picking one “best” segmentation (the two extreme points of the weights choice) one might try to find a set of optimized weights. The STAPLE algorithm [7] widely accepted in the field of medical imaging provides one such approach. It is an expectation-maximization algorithm for simultaneous truth and performance level estimation, where the “true” segmentation is estimated iteratively as a weighted sum of individual segmentations, the weights given by the performance level of each segmenter (algorithm or human), which are in turn estimated from the degree of their agreement with the current estimate of the “true” segmentation. It is important to note that STAPLE relies on the assumption that the individual segmentations are not correlated, which is almost certainly not true for Deep Ensemble predictions, because ensemble models are typically trained on largely the same data. Nevertheless, from the practical point of view we wanted to see how STAPLE performed in our task.

Alternatively, one can train an auxiliary DNN to carry out a nonlinear combination of the ensemble’s segmentations. The original image *I* and a set of all the ensemble’s segmentations $\{S^m\}$ of this image are concatenated into an input image with $m + 1$ channels, and the DNN is trained so that the output image provides the best fit to the ground truth segmentations. We tried both the linear regression and the auxiliary DNN approaches, using the same V-Net architecture for the auxiliary model as for the rest of the study. Large intestine, pancreas and uterus were used for this test. All 16 models in their respective ensembles were used to generate $S^m$ segmentations of the respective training datasets and concatenated with the input images *I* to form a new 17-channel training dataset for the auxiliary DNN.

Figure 4 compares performances of the simple averaging, the linear regression, the auxiliary model solutions, and the STAPLE expectation-maximization approach. The simple averaging was carried out with $p_{thr} = 0.35$. The STAPLE solution also requires some $p_{thr}$ for a binary segmentation mask. $p_{thr} = 0.5$ was used here, but in practice its choice had negligible effects within the 0.05–0.95 range. The error bars indicate one standard deviation of the mean over the test images. One can see that the more sophisticated combinations performed no better than averaging. Probability of the results being equivalent, $p_{null}$, was 0.706, 0.580 and 0.455 in the Kruskal-Wallis test for the large intestine, pancreas and uterus respectively. Hence, simple averaging with $p_{thr} = 0.35$ was used for the rest of the study.

**Fig. 5:**
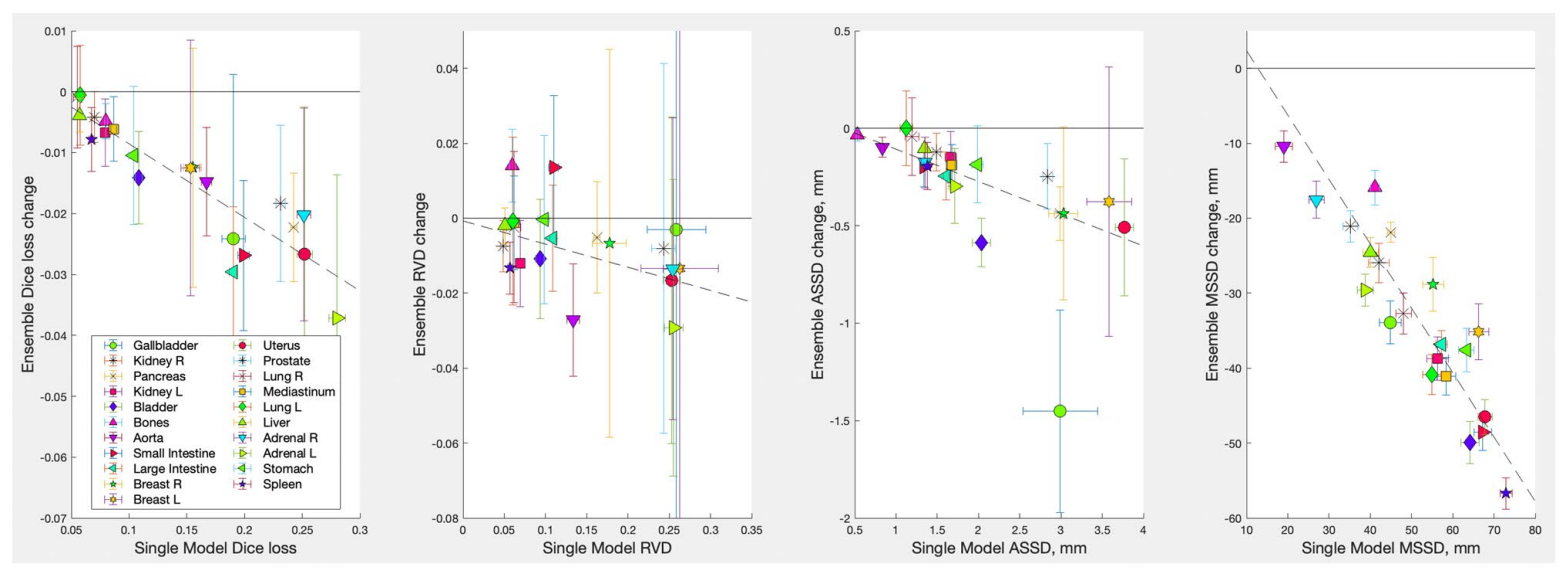
Deep Ensembles gain in performance over an average single model in each ensemble for all organs segmented in this study. The probability threshold, $p_{thr} = 0.35$ was used for all ensemble segmentations. Error bars indicate one standard deviation of the mean over test images; the dashed line shows the linear fit of the gain as a function of an average single model’s performance

**Table 2:** Performance metrics for average single models and Deep Ensembles for all organs segmented in this study. Uncertainties indicate one standard deviation of the mean over test images

**organ**	$DSC_1$	$DSC_{ens}$	$RVD_1$	$RVD_{ens}$	$ASSD_1$	$ASSD_{ens}$	$MSSD_1$	$MSSD_{ens}$
Gallbladder	$0.81 \pm 0.010$	$0.834 \pm 0.025$	$0.259 \pm 0.036$	$0.256 \pm 0.098$	$2.99 \pm 0.45$	$1.54 \pm 0.25$	$44.9 \pm 2.6$	$10.9 \pm 1.1$
Kidney R	$0.93 \pm 0.002$	$0.934 \pm 0.004$	$0.049 \pm 0.003$	$0.041 \pm 0.006$	$1.49 \pm 0.04$	$1.37 \pm 0.09$	$42.1 \pm 2.5$	$16.2 \pm 0.9$
Pancreas	$0.757 \pm 0.003$	$0.779 \pm 0.009$	$0.162 \pm 0.004$	$0.157 \pm 0.014$	$2.99 \pm 0.07$	$2.55 \pm 0.12$	$45 \pm 0.9$	$23.2 \pm 1.0$
Kidney L	$0.92 \pm 0.003$	$0.927 \pm 0.005$	$0.069 \pm 0.005$	$0.057 \pm 0.010$	$1.66 \pm 0.07$	$1.51 \pm 0.11$	$56.3 \pm 2.6$	$17.6 \pm 1.2$
Bladder	$0.891 \pm 0.003$	$0.905 \pm 0.007$	$0.093 \pm 0.005$	$0.082 \pm 0.015$	$2.04 \pm 0.11$	$1.45 \pm 0.06$	$64.2 \pm 2.2$	$14.3 \pm 1.8$
Bones	$0.92 \pm 0.001$	$0.925 \pm 0.003$	$0.060 \pm 0.003$	$0.074 \pm 0.009$	$0.53 \pm 0.01$	$0.495 \pm 0.03$	$41.2 \pm 1.1$	$25.2 \pm 2.1$
Aorta	$0.833 \pm 0.004$	$0.848 \pm 0.008$	$0.134 \pm 0.007$	$0.106 \pm 0.013$	$0.834 \pm 0.03$	$0.737 \pm 0.04$	$19 \pm 2.0$	$8.59 \pm 0.5$
Small Intestine	$0.801 \pm 0.005$	$0.828 \pm 0.011$	$0.110 \pm 0.006$	$0.123 \pm 0.018$	$1.34 \pm 0.04$	$1.14 \pm 0.09$	$67.3 \pm 2.2$	$18.7 \pm 1.2$
Large Intestine	$0.81 \pm 0.003$	$0.84 \pm 0.010$	$0.109 \pm 0.004$	$0.104 \pm 0.014$	$1.61 \pm 0.04$	$1.36 \pm 0.12$	$57.3 \pm 1.4$	$20.5 \pm 1.2$
Breast R	$0.845 \pm 0.008$	$0.857 \pm 0.018$	$0.178 \pm 0.020$	$0.171 \pm 0.048$	$3.03 \pm 0.18$	$2.59 \pm 0.41$	$55.3 \pm 2.5$	$26.4 \pm 2.5$
Breast L	$0.847 \pm 0.008$	$0.859 \pm 0.019$	$0.263 \pm 0.047$	$0.249 \pm 0.112$	$3.58 \pm 0.27$	$3.21 \pm 0.63$	$66.3 \pm 2.4$	$31.1 \pm 2.8$
Uterus	$0.748 \pm 0.007$	$0.775 \pm 0.023$	$0.253 \pm 0.010$	$0.236 \pm 0.042$	$3.77 \pm 0.11$	$3.26 \pm 0.33$	$67.8 \pm 1.7$	$21.3 \pm 1.6$
Prostate	$0.769 \pm 0.004$	$0.787 \pm 0.012$	$0.243 \pm 0.014$	$0.235 \pm 0.047$	$2.84 \pm 0.06$	$2.59 \pm 0.16$	$35.2 \pm 1.8$	$14.1 \pm 1.1$
Lung R	$0.945 \pm 0.004$	$0.946 \pm 0.008$	$0.061 \pm 0.008$	$0.059 \pm 0.018$	$1.2 \pm 0.08$	$1.15 \pm 0.18$	$48.1 \pm 1.9$	$15.4 \pm 2.0$
Mediastinum	$0.913 \pm 0.002$	$0.919 \pm 0.005$	$0.062 \pm 0.005$	$0.061 \pm 0.011$	$1.67 \pm 0.05$	$1.48 \pm 0.09$	$58.4 \pm 2.3$	$17.3 \pm 0.9$
Lung L	$0.942 \pm 0.003$	$0.943 \pm 0.007$	$0.060 \pm 0.009$	$0.059 \pm 0.021$	$1.12 \pm 0.07$	$1.12 \pm 0.18$	$54.9 \pm 2.2$	$14.1 \pm 1.5$
Liver	$0.943 \pm 0.001$	$0.947 \pm 0.003$	$0.051 \pm 0.002$	$0.049 \pm 0.004$	$1.35 \pm 0.03$	$1.24 \pm 0.05$	$40.1 \pm 1.9$	$15.5 \pm 0.6$
Adrenal R	$0.749 \pm 0.006$	$0.769 \pm 0.016$	$0.254 \pm 0.012$	$0.241 \pm 0.038$	$1.35 \pm 0.05$	$1.18 \pm 0.12$	$27 \pm 1.9$	$9.43 \pm 1.7$
Adrenal L	$0.72 \pm 0.007$	$0.757 \pm 0.022$	$0.255 \pm 0.012$	$0.226 \pm 0.038$	$1.71 \pm 0.07$	$1.41 \pm 0.18$	$38.8 \pm 1.9$	$9.21 \pm 0.9$
Stomach	$0.896 \pm 0.004$	$0.907 \pm 0.011$	$0.098 \pm 0.007$	$0.098 \pm 0.021$	$1.99 \pm 0.07$	$1.8 \pm 0.19$	$63.3 \pm 1.9$	$25.7 \pm 2.2$
Spleen	$0.933 \pm 0.002$	$0.941 \pm 0.005$	$0.057 \pm 0.003$	$0.044 \pm 0.006$	$1.38 \pm 0.04$	$1.19 \pm 0.12$	$72.9 \pm 1.5$	$16.2 \pm 1.5$

**Fig. 6:**
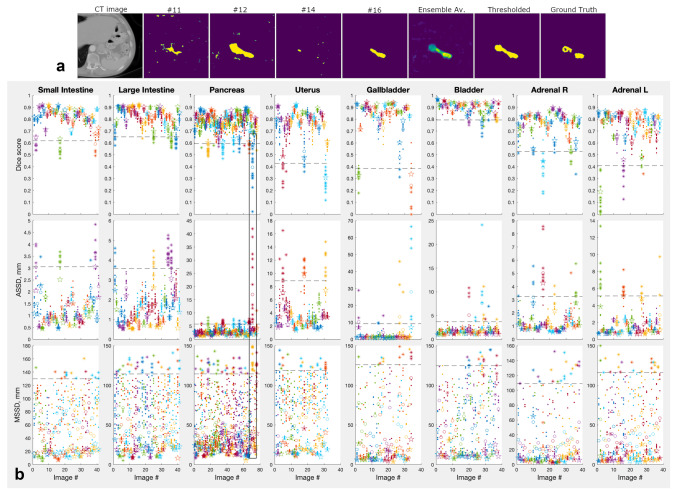
**a** Examples of a “difficult” pancreas image and its segmentations by a Deep Ensemble models #11, #12, #14, and #16, the full 16-model ensemble average, the average thresholded at 0.35, and the ground truth segmentation. The heat map colors vary from blue for organ probability 0 to yellow for organ probability 1. **b** Performance of individual models and their Deep Ensembles for test images of several representative organs. Dots show individual models scores, open circles show averages over the scores; stars show scores for Deep Ensembles comprising the individual models. Dashed lines indicate the outlier boundary for each organ and metric; the outlier data outside of the boundary is plotted with asterisks instead of dots. Data within the black frame corresponds to the images in the top panel

An ensemble’s gain in performance above that of an average single model in the ensemble, $g = L_{ens} - \langle L \rangle$, where *L* stands for one of the loss metrics used here (the Dice loss $DL = 1 - DSC$, *RVD*, *ASSD*, or *MSSD*) is shown in Fig. 5 for all organs segmented (negative *g* values correspond to improved performance). The raw $L_{ens}$ and $\langle L \rangle$ data are presented in Table 2. Except for several outliers, the gains were well fitted with a linear relationship for all metrics and organs. In particular, for the Dice loss the best fit was $g = 0.035 - 0.121 \langle DL\rangle$, and for the *MSSD* the fit was $g = 10.94 - 0.86 \langle MSSD\rangle$, i.e., on average, an ensemble improved a single model’s performance by $12.1 \pm 0.3$% for the Dice loss and $86 \pm 5$% for the MSSD metric. For the *RVD* and *ASSD* metrics the improvements were $6.2 \pm 0.7$% and $16.6 \pm 0.4$% respectively. The fit coefficients and their standard deviations were obtained using the York algorithm applied to loss metrics *L* and their standard deviations represented by x and y error bars in Fig. 5 as described in [39]. One can see that ensembles improved the volume error *RWD* only moderately. In contrast, the maximal location error *MSSD* improved quite dramatically. This indicates that the main contribution of the ensembles was to reduce the occurrence of spurious outlier regions in segmentations. Examples of such outlier regions are displayed in Fig. 6a.

### Deep Ensembles Prevent Bad Failures Characteristic of Single Models

So far the results were focused on the boost in the average segmentation performance achieved by Deep Ensembles. In the context of medical images there is another aspect, possibly, even more important than an algorithm’s average performance: its robustness. Because neural networks are to a large degree black boxes, there is always a possibility of a catastrophic failure in a solution, which can lead to a costly medical error. It was argued that combining diverse solutions by means of the majority vote or a similar scheme should result in a more reliable solution, because diverse solutions are likely to have independent failure points [11, 19]. By implementing a wide combination of solutions Deep Ensembles fit the bill [40]. We tested if they were indeed effective in preventing catastrophic failures resulting in very bad segmentations.

Figure 6a illustrates the difficulties that individual models can encounter in segmenting some images, and the degree of variation among identical models trained on identical data starting from random weights. From left to right: an axial slice of a 3D volume containing the pancreas, its segmentations by several Deep Ensemble’s models, the average of all ensemble segmentations (16 models), the average thresholded at $p_{thr}=0.35$, and, finally, the ground truth segmentation. Figure 6b plots Dice scores, *ASSD* and *MSSD* metrics (*RWD* was omitted to reduce clutter) of individual models and their ensembles for every test image of several organs, where bad failures of individual models were most common. The first thing to notice is the large variation among individual models performances for a given image (shown with dots), which is particularly striking for those CT images, where the task was the hardest (e.g., the image in panel a). The individual variations are especially pronounced for the MSSD metric.

Some models showed catastrophic failures for certain images, even though on average such models performed on par with other models. We define a “catastrophic failure” of a given model on a given image as an event where any of the segmentation metrics used here produced an outlier compared to the same metric applied to the rest of the images for the same organ. Outliers were defined as *DSC* values below the 5% percentile and the *RVD*, *ASSD*, *MSSD* values above their 95% percentiles. The corresponding outlier boundaries were shown with dashed lines, and the outlier data (data beyond the boundary) were plotted with asterisks in Fig. 6b. One can see that Deep Ensembles (results shown with stars) largely avoided catastrophic failures in the sense that their metrics were mostly on the “safe” side of the outlier boundary. To quantify this we defined the “high risk images”: images for which at least one model produced an outlier metric. These images comprised about 12% of the test images across all organs. Ensembles performed without outliers for 83%, 68%, 90%, and 100% of the “high risk images” when measured in terms of the *DSC*, *RVD*, *ASSD* and *MSSD* metrics respectively.

**Fig. 7:**
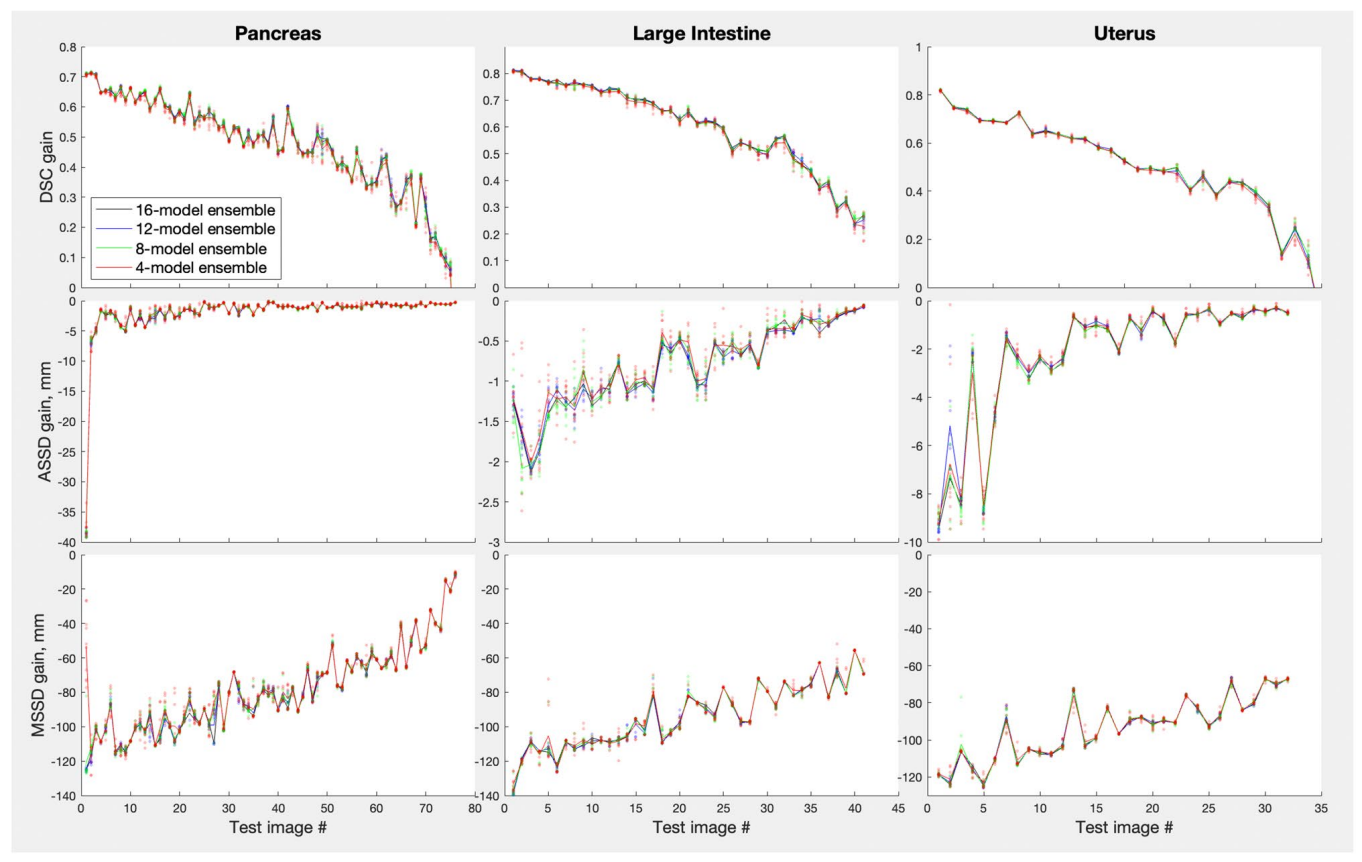
Gain in performance of Deep Ensembles over the worst model in the ensembles for large intestine, pancreas, and uterus. Results for different ensemble sizes are presented by different colors, the ensemble sizes 12, 8, and 4 were created by randomly subsampling the required number of models from the full 16-model ensembles repeated ten times. Semi-transparent dots show such subsampled ensembles data, while lines show average performance over the 10 subsampled ensembles. Test images were ordered along the x-axis from the poorest to the best performance for each metric

Given that on the organ level ensembles helped the most for those organs where single models struggled the most (Fig. 5) one might ask if the same is true on the individual image level. Similarly to Figs. 5 and 7 plots ensemble gains over a single model, $g = L_{ens} - L_{worst}$, where *L* stands for one of the loss metrics used here, but in this case metric $L_{worst}$ for the worst model is used as a reference for each test image instead of the average single model metric $\langle L \rangle$. The *RVD* metric was omitted to reduce cluster, the same trends were observed for this metric as for the rest. The images were sorted so that the worst-metric image was the first on the x-axis and the best-metric image was the last. Plotted this way, one can see a clear trend in the data: the poorer is a single model performance for a given image, the larger is the ensembling gain for that image (probability of the null trend, $p_{null}$, in the Kruskal-Wallis test was extremely low for all the datasets shown). Figure 7 demonstrates other important points. (i) Small ensembles (size 4, red color) were almost as effective in improving single-model failures as large ensembles (size 16, black color). (ii) Ensembles themselves did not fail on any images: there was very little variation in performance when smaller ensembles were randomly subsampled from a large ensemble. This can be seen as the very tight spreads of the semi-transparent dots around the solid lines showing their averages (with the possible exception of the smallest ensembles, size 4, red color). In other words, ensembles prevented failures of individual models robustly.

### Stochastic Weight Averaging

The performances of the SWA-trained models were very similar to those shown in Fig. 6 and we did not plot them. While for some models the SWA procedure improved the final Dice Score by several percent, for others it made it worse, the net effect was an improvement of less than 0.1%. The Deep Ensemble of the SWA models had a similarly tiny gain over the ensemble of the original models. Importantly, the same occasional catastrophic failures were observed for SWA models as for the original models illustrated in Fig. 6. This demonstrates that the failures resulted not from a poor approximation of the “true” local optimum due to a limited training set, but from the optimum occasionally being a really poor fit for the new image. Hence, exploiting multiple local minima of the loss function appears to be instrumental in preventing such failures, and no locally ensembling schemes like SWA can achieve it.

### Distillation of Deep Ensembles

**Fig. 8:**
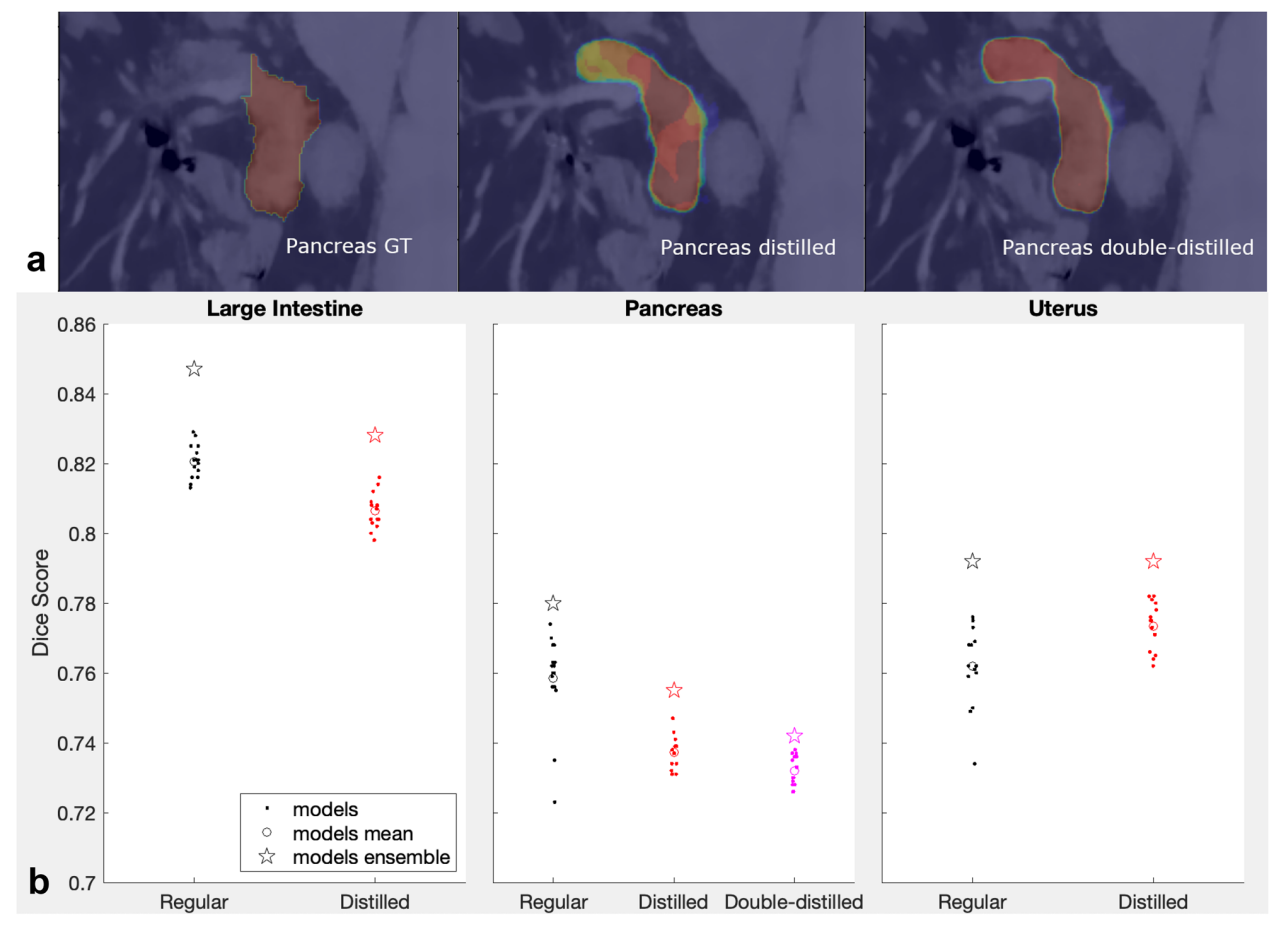
**a** Examples of the pancreas segmentations (a representative single slice is shown). Left to right: original ground truth, single-distilled “ground truth”, double-distilled “ground truth”. **b** Results of distillation of Deep Ensembles for the large intestine, pancreas and uterus. For the pancreas the second distillation results were also plotted. Dice scores for individual models are shown with dots, their averages — with open circles, the ensembles Dice scores — with stars

Examples of the ground truth segmentations used for training distilled models are shown in Fig. 8a for the pancreas. Distillation results for the large intestine, pancreas, and uterus are shown in Fig. 8b. The performances of all models (original, distilled, double-distilled, and their Deep Ensembles) were compared by calculating Dice scores of the respective segmentations over the true ground truth segmentations. Distillation proved to be rather ineffective for organ segmentations: except for the uterus results, Dice scores of distilled models (red dots) were on average below Dice scores of the original models (black dots, model averages shown with open circles) with probabilities $p_{null}$ of them coming from the same distribution less than 1e-3 in the Kruskal-Wallis test. Furthermore, the double-distilled models (magenta dots) for pancreas were performing poorer than single-distilled models, $p_{null}$ = 0.0037 in the Kruskal-Wallis test.

**Fig. 9:**
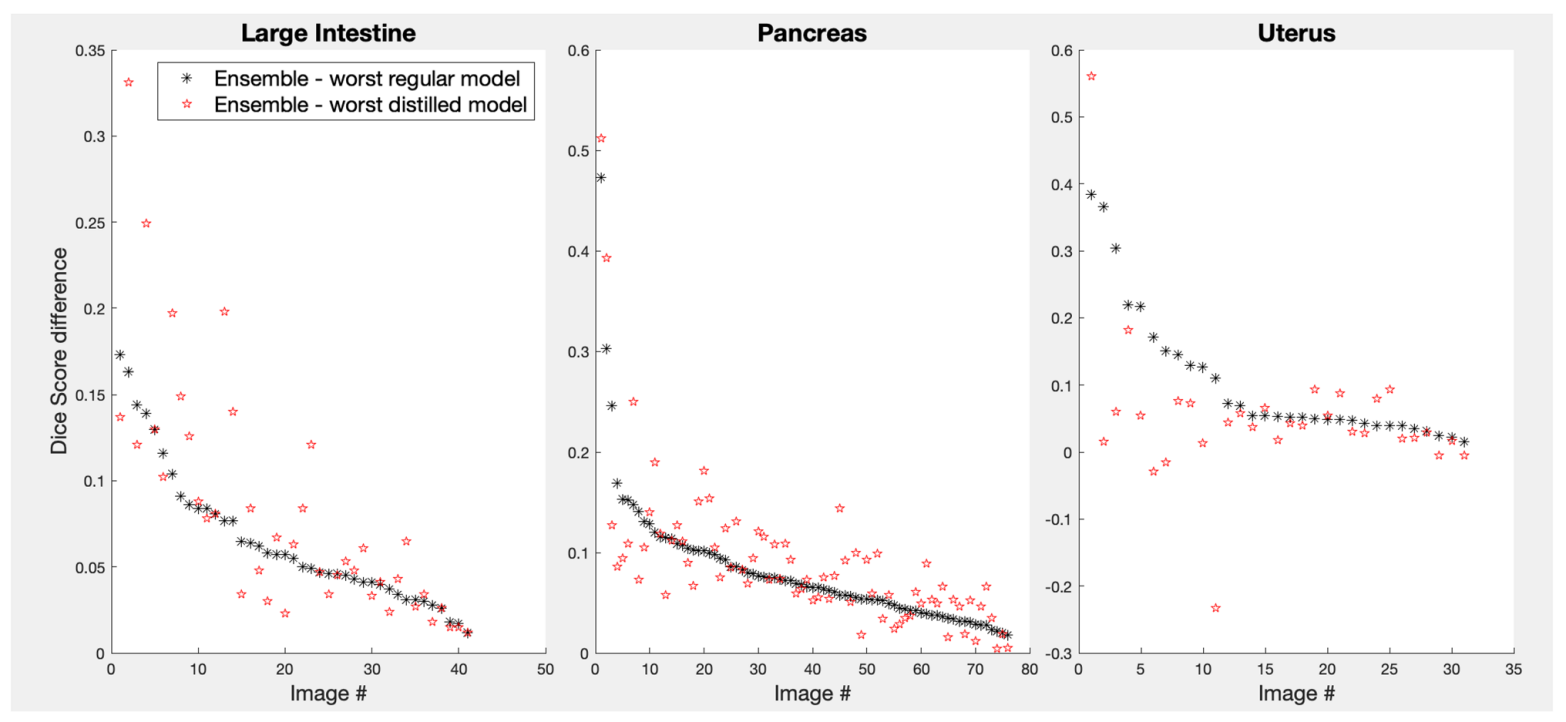
Gains in performance of 16-model Deep Ensembles over the worst model in the ensembles (black asterisks) and over the worst *distilled* model for the same ensembles. The test images were ordered along the x-axis from the highest to the lowest gain over the regular models performance

As mentioned above, the ability of Deep Ensembles to find more robust solutions is, arguably, more important than being able to find solutions with higher average performances. Distilled models did not perform better than regular models, but could they still be more robust? Black dots in Fig. 9 plot differences of Dice scores between Deep Ensembles (16 models) and the worst-performing model in each ensemble for the three organs for which ensemble distillations were performed. For illustrative purposes, indices of the images plotted along the x-axis were sorted in such a way that the differences were ordered from highest to lowest. The red stars show gains of the same Deep Ensembles, but over the worst *distilled* model instead. If the distilled models were more robust than the regular models, the red symbols (ensemble gains) would appear below the respective black symbols. In fact, on average, worst distilled models performed on par with worst regular models, and even for the uterus models, where distillation improved the average performance, their worst results were often poorer than those for regular models.

## Discussion

We found that simple averaging of the Deep Ensemble solutions was as effective as more sophisticated averaging schemes in boosting segmentation accuracy. This is in fact how Deep Ensembles are commonly used and in agreement with another segmentation study, where a different set of trained combiners (weighted majority vote, Naive Bayes and Behaviour Knowledge Space (BKS)) were tried on liver ensembles [13]. On average, ensembles reduced the Dice loss (the gap between a single model’s performance and 100%) by about 12%. This is similar to the improvements found for image classification on CIFAR 10 and ImageNet datasets [40]. The Hausdorff distance from the ground truth segmentation decreased much more: by 86%, which indicates that ensembles effectively prevented outlier regions from being included into the segmentation. The ensembling benefit quickly saturated about 8–10 models per ensemble, which was very similar to the way ensemble size affected performance for classification tasks [40]. Unlike classification tasks, the segmentation task also required to the choice of a threshold to obtain the binary segmentation mask from the average. Given the known ground truth segmentations, we found that the threshold value of 0.35 maximized Dice scores on average for various organs in this study. Varying this value from 0.25 to 0.5 produced only marginal changes to performance metrics, so the precise value within this range was not critical. However, a choice of the threshold value outside of this range may still be used to shift the balance between false positives and false negatives in the ensemble predictions.

The main novel finding of this study was that while single models were prone to unexpected bad failures in segmenting some images, Deep Ensembles were robust to such events and improved the single-model performance the more the model struggled for a given image. This makes Deep Ensembles particularly valuable in situations where the cost of error is high, including various medical applications.

The ineffectiveness of the distillation found in our study differs from results for classification tasks, where the distilled models were reported to perform better than the original models and about as well as the ensembles they were derived from [36, 41]. This discrepancy can be explained by the fact that training accuracy of recent models for standard classification datasets (e.g., ResNet trained on CIFAR-10/100) is nearly 100%, which makes distilled training data labels almost identical to the true labels, except for some added “softness”. In our case, Dice scores rarely reached 95% during training for “difficult” organs, such as the ones shown in Fig. 8. This makes segmentations used for distillation training not only “soft”, but also somewhat incorrect, as can be seen comparing the ground truth pancreas segmentation in Fig. 8a with its distilled and double-distilled versions.

Notably, the same as with the original models, the distilled models did not converge to the same solution/local minimum: although their Dice scores generally varied somewhat less than the original ensemble models scores, they still varied. The variation appears to be further reduced for the double-distilled models of the pancreas, and the average Dice score for the double-distilled models was even lower than that for the single-distilled models. The same was true for their ensembles. In the original distillation paper, authors argue that it is the “soft” property of labels incorporating not only the truth, but also the errors of the model that make the distillation training beneficial [36]. This point is further expounded in [41], which explains the distillation benefits by pushing the distilled model to fit soft ensemble’s labels and, in this way, necessarily learn all the features that the ensemble has learned. One might also argue that distillation does its trick by reshaping the actual ground truth into something that has more “affinity” to the model itself, i.e., into something that the model can actually fit well. This modification probably regularizes the loss energy landscape into something having a better-defined global minimum or, at least, a global depression with a multitude of local minima scattered over it. This would explain the bunching-up of Dice scores observed when moving from the original ensemble to the single-distilled ensemble, to the double-distilled ensemble in Fig. 8.

The fact that distilled models do not appear to be more robust than regular models suggests that the advantage of Deep Ensembles is not merely in aggregating learned features across the constituent models, because in this case distilled models would have had a comparable robustness. Along with features relevant to the task, lots of irrelevant features are learned by each model. These irrelevant features capture accidentals in the dataset and lead to overfitting, the more so the smaller the training dataset is. The irrelevant features can occasionally be present in an image in such abundance, that erroneous segmentations are made. Examples of such random-looking segmentations, with pixel-level probabilities close to 1, can be seen in Fig. 6a. However, because the learned irrelevant features are likely to be uncorrelated among the Deep Ensemble models, the erroneous segmentations that they occasionally produce would not be correlated either and effectively average out once all ensemble’s segmentations are averaged and thresholded. This is illustrated in the last three panels of Fig. 6a. Without averaging and thresholding over multiple segmentations the irrelevant features might still manifest themselves as occasional bad segmentations, even if all the relevant features have been learned by a distilled model. This explains why distillation to a single model can improve the performance almost to the level of the whole ensemble (if all the aggregated relevant features were captured by the distillation), but does not necessarily make the model as robust as the whole ensemble: due to the input of the irrelevant features, a single model is likely to fail badly sooner or later.

The V-Net model architecture used in this study along with a very similar U-Net architecture are the most common architectures used for image segmentation. It is possible that the findings reported here will not hold when ensembling DNN models of other architectures. While we observed that simple averaging of models solutions works as well or better than more sophisticated ensembling schemes (other linear and nonlinear combinations of the solutions as well as averaging of the weights for the SWA ensembles), it is still possible that some other ensembling approach will surpass the benefits of the simple averaging. For example, one could attempt to find optimal combinations of the ensemble models along the high-accuracy pathways in the weight space as suggested in [42].

## Conclusions

In this study, we investigated the use of Deep Ensembles for organ segmentation in CT images. Simple averaging of model predictions in Deep Ensembles, followed by thresholding, gave the same increase in Dice scores as more sophisticated combination methods. We found that the thresholding probability 0.3–0.4 resulted in the best performance overall. On average, using ensembles reduced the Dice loss (the gap between a single model’s Dice score and 100%) by about 12%. The Hausdorff distance from the ground truth segmentation decreased by 86%, which testifies that ensembles effectively exclude outlier regions from segmentations. Distillation of Deep Ensembles did not consistently produce higher performing single models. Most importantly, our study showed that while single models (distilled models included) can be expected to show occasional bad failures for some images, Deep Ensembles are robust to such events. This makes Deep Ensembles particularly well suited for the applications, where the cost of error is high, including medical applications.

## Data Availability

The in-house datasets may not be made publically available due to the Genetech Inc. internal regulations of clinical data usage.
